# Serum Brain-Derived Neurotrophic Factor Levels in Different Neurological Diseases

**DOI:** 10.1155/2013/901082

**Published:** 2013-08-19

**Authors:** Mariacarla Ventriglia, Roberta Zanardini, Cristina Bonomini, Orazio Zanetti, Daniele Volpe, Patrizio Pasqualetti, Massimo Gennarelli, Luisella Bocchio-Chiavetto

**Affiliations:** ^1^Department of Neuroscience, AFaR—Fatebenefratelli Hospital, Isola Tiberina 39, 00186 Rome, Italy; ^2^Neuropsychopharmacology Unit, IRCCS “S. Giovanni di Dio” Fatebenefratelli, Via Pilastroni 4, 25125 Brescia, Italy; ^3^Alzheimer's Disease Unit, IRCCS “S. Giovanni di Dio” Fatebenefratelli, Via Pilastroni 4, 25125 Brescia, Italy; ^4^Parkinson's Rehabilitation Unit, AFaR-Fatebenefratelli S. Raffaele Arcangelo Hospital, Madonna dell'Orto 3458, 30121 Venice, Italy; ^5^Medical Statistics & Information Technology, AFaR—Fatebenefratelli Hospital, Isola Tiberina 39, 00186 Rome, Italy; ^6^Division of Biology and Genetics, Department of Molecular and Translational Medicine, University of Brescia, Viale Europa 11, 25123 Brescia, Italy; ^7^Genetic Unit, IRCCS “S. Giovanni di Dio” Fatebenefratelli, Via Pilastroni 4, 25125 Brescia, Italy

## Abstract

Consistent evidence indicates the involvement of the brain-derived neurotrophic factor (BDNF) in neurodegenerative disorders such as Alzheimer's disease (AD) and Parkinson's disease (PD). In the present study, we compared serum BDNF in 624 subjects: 266 patients affected by AD, 28 by frontotemporal dementia (FTD), 40 by Lewy body dementia (LBD), 91 by vascular dementia (VAD), 30 by PD, and 169 controls. Our results evidenced lower BDNF serum levels in AD, FTD, LBD, and VAD patients (*P* < 0.001) and a higher BDNF concentration in patients affected by PD (*P* = 0.045). Analyses of effects of pharmacological treatments suggested significantly higher BDNF serum levels in patients taking mood stabilizers/antiepileptics (*P* = 0.009) and L-DOPA (*P* < 0.001) and significant reductions in patients taking benzodiazepines (*P* = 0.020). In conclusion, our results support the role of BDNF alterations in neurodegenerative mechanisms common to different forms of neurological disorders and underline the importance of including drug treatment in the analyses to avoid confounding effects.

## 1. Introduction

Neurological disorders have rapidly become a significant and growing problem, affecting more than 450 million individuals worldwide. The most common forms of neurodegenerative diseases include Alzheimer's disease (AD), Parkinson's disease (PD), frontotemporal dementia (FTD), vascular dementia (VAD), and dementia with Lewy bodies (LBD).

The identification of molecular dysregulations associated with the diagnosis of neurodegenerative disorders may provide important information for the clarification of the pathogenetic mechanisms and for the discovery of new biomarkers for an early, accurate, and differential diagnosis of these diseases [1].

Recently, there has been increasing evidence that alterations in the brain neurotrophic support and in particular in the brain-derived neurotrophic factor (BDNF) expression and signaling might contribute to neurodegeneration [2]. The BDNF is a member of the neurotrophin family of proteins that is not only important for the normal development of the peripheral and central nervous system but also plays a key role in neuronal survival and synaptic plasticity in the adult brain [3]. Altered functionality of BDNF has been observed in different neurodegenerative diseases [4, 5]. A reduction of BDNF mRNA and protein expression has been consistently reported in multiple brain areas of AD postmortem and in the substantia nigra of PD patients [6, 7]. Limited information is available for non-AD forms of dementia, although a neurotrophin decrease was observed in LBD but not in FTD patients [8, 9]. Impairments in BDNF synthesis have been associated with the hallmarks of neurodegenerative pathogenesis [10]: a lack of BDNF has been selectively observed in neurons containing neurofibrillary tangles in AD [11], and a loss of BDNF production has been associated with mutations of alpha-synuclein in early-onset familial PD [12].

BDNF is also present in large quantities in blood platelets [13]. Despite the name “brain-derived,” many peripheral sources, among them epithelial and vascular cells, muscle cells, macrophages, and leucocytes [14–16], may synthesize and release BDNF. The possibility of bidirectional transit of the neurotrophin across the blood-brain barrier is still an open area of investigation [17, 18]. Nevertheless, evidence does suggest that serum BDNF levels may reflect what occurs in the brain since in recent years, the involvement of BDNF in the pathogenesis of several mental disorders has been corroborated by a series of biochemical studies. In particular, reduced BDNF serum concentrations were consistently observed in several adulthood psychiatric pathologies such as major depression disorder [19, 20]. Furthermore, in healthy subjects, correlations were observed between serum BDNF levels and cerebral cortex integrity in neuroimaging studies [21] and in neuropsychological performances [22].

Peripheral BDNF levels have been analyzed in AD and PD patients reporting contrasting results. While some researchers have found a decrease in serum concentrations in AD and PD subjects [23–26], other studies have shown increased BDNF levels in patients in the early phases of AD [23, 27] and in patients with advanced AD [28]. One study found a decrease in serum BDNF in AD patients but did not show significant differences in VAD patients [29]. To date, no studies have investigated alterations of neurotrophin blood levels in other forms of neurodegenerative diseases, such as FTD or LBD. One possible explanation for the discrepancies in BDNF biochemical markers in the blood might be the possible effect of drug treatments in patients with neurodegenerative disorders. In fact, many drugs employed in the treatment of these pathologies, such as acetylcholinesterase inhibitors, anti-Parkinsonian agents, or antidepressants, may modulate BDNF expression [30–33].

In order to contribute in the clarification of the involvement of BDNF in neurodegenerative disease, replicating and extending previous findings, in this study we have compared BDNF serum levels in a large sample of patients affected by different neurological diseases and in a group of healthy participants. Moreover we have analyzed possible modifications induced by the most frequent drug treatments in neurodegenerative disorders to evaluate potential confounding effects on BDNF measures.

## 2. Material and Methods

### 2.1. Participants

The sample consisted of 624 participants: 169 controls, 266 AD, 28 FTD, 40 LBD, 91 VAD, and 30 idiopathic PD patients. Patients with AD, FTD, LBD, VAD, and control subjects were enrolled at the Alzheimer Unit of IRCCS-Fatebenefratelli (Brescia, Italy); PD patients were recruited at the Rehabilitation Unit, Hospital San Raffaele Arcangelo, Fatebenefratelli (Venezia, Italy). The study protocol was approved by the local ethics committees, and written informed consent was obtained from each individual prior to participation in the study. All patients were examined by neurologists with expertise in neurodegenerative diseases and met the internationally standardized criteria [34–39] for dementias or PD disorder. The severity of cognitive impairment was assessed by Mini-Mental State Examination scores (MMSE) [40]; staging in patients with PD was recorded with Hoehn and Yahr scores [41]. The majority of the patients involved in this study received pharmacological treatment for their illnesses and often took more than one medication simultaneously. Demographic and clinical features of the sample and the number of patients taking each of the more frequent classes of psychotropic medications (neuroleptics, benzodiazepines, antidepressants, mood stabilizers/antiepileptics, and cholinesterase inhibitors) are shown in Table 1. All PD patients received treatment with L-DOPA.

Control participants were recruited according to the following criteria: absence of alcohol, substance abuse, and relevant neurological diseases, MMSE scores of 27/30 or higher, and a negative personal and family history (first-degree relatives) of psychiatric DSM-IV Axis I disorders (confirmed with the Mini-International Neuropsychiatric Interview M.I.N.I).

### 2.2. Biochemical Investigation

Venous blood (5 mL) was collected from each patient in the morning (between 8 and 9 a.m.) after overnight fasting. Blood samples were collected into tubes without anticoagulant and allowed to clot at room temperature for 1 h. Serum was separated by centrifuge at 1620 rcf for 15 min and then stored at −80°C until assay. BDNF levels were measured by the ELISA method using the human BDNF Quantikine kit (R&D system, Minneapolis, USA), according to the manufacturer's instructions. BDNF content was expressed as the equivalent of the human recombinant protein. The detection limit was 20 pg/mL, and data were expressed as ng of protein/mL of serum. All samples and standards were measured in duplicate. Samples of patients with different diagnoses and control participants were analysed together in the ELISA templates, and possible variability between different assays was controlled using 4 samples of known BDNF concentration. The mean interassay precision, expressed as the coefficient of variation (%), was about 8%.

## 3. Statistical Analysis

Statistical analyses included *T*-test, chi-squared test, analysis of variance (ANOVA) with post-hoc pairwise Bonferroni analysis, analysis of covariance (ANCOVA), and calculations of Pearson's linear correlation coefficients when appropriate. Significance for the results was set at *P* < 0.05. SPSS, version 17.0 (http://www.spss.com/), was used for all statistical calculations.

## 4. Results

**Figure 1:**
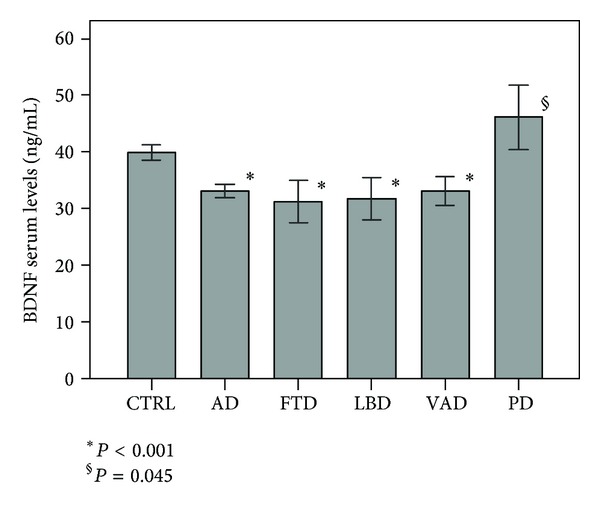
BDNF serum levels (mean, 95% CI) in patient and control samples (*P* values are referred to comparisons versus controls).

Demographic characteristics for controls and patients are reported in Table 1. No effects of age and sex variables on serum BDNF were observed in the control group: despite a large age range (min = 19, max = 88, SD = 15.7) and a balanced sex distribution (49% F, 51% M), only 0.3% of BDNF variability was accounted for by age (*P* = 0.316) and 1.3% by sex (*P* = 0.137). However, significant differences were observed in age and sex distributions between patients and controls in our sample (Table 1). Thus, possible confounding effects of these variables were controlled by including them as covariates in all statistical analyses. BDNF serum levels (Table 1, Figure 1) in the controls and in the patient groups were compared by one-way analyses of variance (ANOVA), and significant differences were found (F(5,621) = 17.41, *P* < 0.001). Post-hoc analyses, using Bonferroni correction, found significant decreases in serum BDNF levels in AD, FTD, LBD and VAD patients compared with controls (*P* < 0.001). On the contrary, significant increases in serum BDNF levels were observed for PD patients (*P* = 0.045). All differences remained statistically significant after ANOVA analyses for sex and age variables.

No correlation between BDNF serum levels and cognitive impairments, as measured by scores on the MMSE, were observed in any dementia groups (*P* > 0.20) (PD patients are not included in this analysis). Analogously, we found no correlation between BDNF serum levels and PD symptoms, as measured with the HY scale (*P* = 0.45).

**Figure 2:**
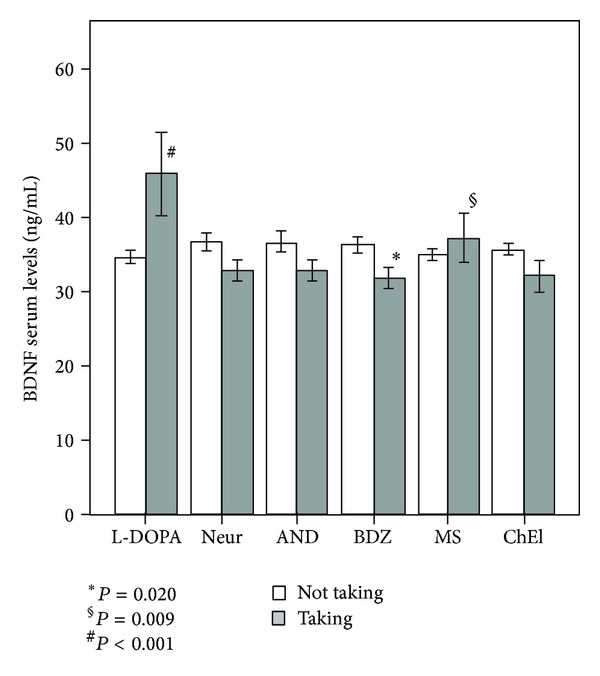
BDNF serum levels (mean, 95% CI) in patients taking (grey) and not taking (white) different psychotropic drugs (neuroleptics: Neur, benzodiazepines: BDZ, antidepressants: AND, mood stabilizers/antiepileptics: MS, cholinesterase inhibitors: ChEI and L-DOPA).

Analyses of potential modulatory effects of pharmacological treatments (Table 2, Figure 2) on BDNF levels in the whole sample of patients taking various common psychiatric medications revealed higher BDNF serum levels in patients treated with mood stabilizers/antiepileptics (*P* = 0.009) and L-DOPA (*P* < 0.001) and significant reductions in neurotrophin concentrations in patients treated with benzodiazepines (*P* = 0.020). Differences in BDNF serum levels in demented patients toward controls remained significant after correction for the presence of pharmacological treatment with mood stabilizers/antiepileptics and benzodiazepines. Since all PD patients were administered L-DOPA and none of the other patients were taking such drug, the confounding effect could not be solved, and we could not assess whether the BDNF increase in PD patients was due to L-DOPA medication.

## 5. Discussion

The principal objective of our work was to evaluate putative alterations of BDNF serum levels in patients affected by neurodegenerative disorders and possible applications of the serum dosage as a biochemical marker for differential diagnosis. Our results showed a decrease in BDNF serum levels common to the different forms of dementias and an increase in the neutrophin content in patients affected by PD.

In accordance with our results, decreased serum concentrations of BDNF have been described in patients affected by AD [23, 24, 29, 42], although some studies have reported no differences [28, 42] or even increases in neurotrophin levels [27]. These discrepancies might be explained, at least in part, by differences in the severity of the cognitive impairments associated with the different AD samples. In fact, increased serum BDNF levels have been observed in patients with MCI and early AD [27, 42], supporting a possible compensatory augmentation of neurotrophin synthesis in the earliest stages of AD progression. Our sample was instead composed of AD patients with a moderate/severe degree of cognitive impairment (MMSE 13.6 ± 8.0), and no associations were observed between illness severity and BDNF serum levels in our sample. We observed for the first time lower serum BDNF levels in VAD, LBD, and FTD patients, indicating a generalized reduction of the neurotrophin in dementia. Our data on VAD contrast with negative findings reported by Yasutake and colleagues [29]. Again, in this case, the inconsistency in reported results might be ascribed to the advanced cognitive impairment of our sample (MMSE 14.8 ± 8.0).

Overall, the results of our study limited the usefulness of BDNF as a biomarker for specific forms of dementias and, instead, support the involvement of BDNF in common pathogenetic mechanisms in all cognitive disorders analyzed. The BDNF pathway has been consistently linked to core pathological features of amyloidosis and tauopathies because this trophic factor is able to regulate the production and the neurotoxic effects of amyloid b42 and Tau protein hyperphosphorylation [43, 44]. Furthermore, BDNF can elicit neuroprotective effects on neurons after vascular damage [45]. In general, BDNF seems to be essential to the survival and the functioning of mature, fully developed neurons in the adult brain, and perturbations in neurotrophin expression and trafficking have been associated with a wide spectrum of neurological and psychiatric disorders [46]. Interestingly, reduced serum BDNF levels have also been associated with worse cognitive performance in healthy elderly subjects [22], suggesting that variations in BDNF concentrations might also indicate a progressive shift toward a neurodegenerative state in the healthy elderly [47]. Finally, we found an increase in serum BDNF levels in PD patients, in partial disagreement with a recent study reporting decreased levels of BDNF in patients in an early stage of PD disease and with a moderate severity of motor function impairment [26]. PD patients in our study had severe motor impairments. Scalzo and colleagues also reported higher serum BDNF levels in patients in advanced stages of the disease. Moreover, the observed increase in BDNF in PD patients in our study could be explained by compensatory effects of pharmacological treatment with L-DOPA, whose increase might be viewed as a neuroprotective mechanism. Treatment with L-DOPA induces BDNF release from corticostriatal fibers, and this mechanism consecutively enhances the expression of D3 receptors and maximizes the beneficial symptomatic effects of treatment [48].

These observations underline the necessity of controlling for the presence of drug treatment in biochemical studies of serum BDNF. Patients with dementias and PD are treated with a plethora of psychiatric drugs, ranging from cholinesterase inhibitors and L-DOPA used to treat core symptoms to antidepressants, neuroleptics, mood stabilizers and benzodiazepines used to manage the behavioral and psychotic symptoms that often accompany these diseases [49]. Numerous clinical studies evidenced the effect of medication, including antidepressants, mood stabilizers and atypical antipsychotics [32, 50, 51] or lithium and cholinesterase inhibitors [23, 30] on the BDNF concentrations in neuropsychiatric diseases. In this study, significantly lower concentrations of BDNF were observed in patients treated with benzodiazepines (*P* = 0.020). The relationship between BDNF levels and benzodiazepines has been already investigated in other neuropsychiatric disorders. Diazepam was reported to reduce levels of BDNF [52] in animals, and, more recently, decreased levels of serum BDNF have been observed in schizophrenia patients with catatonia treated with lorazepam [53]. This association would seem to be linked to the known effect of BDNF on the GABA neurotransmitter [54–56] responsible for the sedative and anxiolytic effects of benzodiazepines. Moreover, an increase in BDNF levels was observed in patients taking a mood stabilizer that is in accordance with the hypothesis [57] that these drugs play an essential role in regulating BDNF expression. However, results obtained from studies of peripheral BDNF serum changes induced by these therapeutic drugs are not always consistent [58, 59] and may depend not only on the pharmacological characteristics of the drug but also on length of treatment [60].

Except for L-DOPA, for which the effect couldn't be discriminated, the differences in BDNF levels in our work remained significant for all dementias after correcting for the effect of various pharmacological treatments.

This study presents some limitations related mainly to the different size of the sample in fact for some pathologies under study the number of cases analyzed was lower than others. In addition, many of the patients recruited were in multitherapy, and this is certainly a further confounding factor for the interpretation of results.

## 6. Conclusions

In conclusion, our findings support the hypothesis that BDNF alterations are involved in neurodegenerative mechanisms and suggest that low BDNF levels are nonspecific markers of neurodegeneration common to several cognitive disorders. Our study also underlines the importance to control for treatment with psychotropic drugs in all analyses of BDNF to avoid confounding effects. Indeed, our results provide further information about the involvement of BDNF in dementias and in PD for the implementation of new therapeutic strategies.

## Figures and Tables

**Table 1:** Demographic, clinical features, and BDNF serum levels in different study groups.

Diagnosis	CTRL	AD	FTD	LBD	VAD	PD
Sample size	169	266	28	40	91	30
Demographic variables						
Gender (% F)	49%	67%	54%	68%	67%	30%
differences versus CTRL *P* =		<0.001	0.688	0.052	0.006	0.073
Age	48.0 ± 15.7	80.1 ± 7.1	71.7 ± 9.9	79.2 ± 5.6	81.9 ± 7.5	67.6 ± 8.4
differences versus CTRL *P* =		<0.001	<0.001	<0.001	<0.001	<0.001
Clinical variables						
MMSE score	>27	13.6 ± 8.0	16.1 ± 6.6	15.9 ± 7.4	14.8 ± 8.0	25.4 ± 2.0
HY score						3.58 ± 0.5
Pharmacological treatment						
Neuroleptics (−/+)*		118/148	14/14	14/26	44/47	26/44
Benzodiazepines (−/+)		167/99	17/11	28/12	58/33	30/0
Antidepressants (−/+)		142/124	12/16	20/20	27/64	17/13
Mood stabilizers antiepileptics (−/+)		237/29	20/8	37/3	74/17	30/0
Cholinesterase inhibitors (−/+)		193/73	27/1	21/19	87/4	30/0
L-DOPA (−/+)		266/0	28/0	40/0	91/0	0/30
Serum BDNF						
ng/mL (mean ± SD)	39.89 ± 9.48	33.16 ± 12.4	31.19 ± 9.68	31.71 ± 11.6	33.06 ± 12.4	46.13 ± 15.3

*(−/+): not treated/treated.

**Table 2:** BDNF serum levels (mean ± SD) in patients taking (+) and not taking (−) different psychotropic drugs.

Pharmacological treatment	*N*	BDNF ng/mL
Neuroleptics (+)	239	33.06 ± 10.65
Neuroleptics (−)	216	34.50 ± 12.15
Benzodiazepines (+)	155	32.02 ± 9.81
Benzodiazepines (−)	300	34.64 ± 12.06
Antidepressants (+)	237	33.14 ± 11.05
Antidepressants (−)	218	34.40 ± 11.76
Mood stabilizers antiepileptics (+)	57	37.43 ± 12.32
Mood stabilizers antiepileptics (−)	398	33.21 ± 11.18
Cholinesterase inhibitors (+)	97	32.36 ± 10.64
Cholinesterase inhibitors (−)	358	34.12 ± 11.58
L-DOPA (+)	30	46.13 ± 15.30
L-DOPA (−)	425	32.87 ± 10.56
